# Maillard-Type Protein–Polysaccharide Conjugates and Electrostatic Protein–Polysaccharide Complexes as Delivery Vehicles for Food Bioactive Ingredients: Formation, Types, and Applications

**DOI:** 10.3390/gels8020135

**Published:** 2022-02-21

**Authors:** Xiaohong Sun, Hao Wang, Shengnan Li, Chunli Song, Songyuan Zhang, Jian Ren, Chibuike C. Udenigwe

**Affiliations:** 1 College of Food and Biological Engineering, Qiqihar University, Qiqihar 161006, China; xs7@ualberta.ca (X.S.); wh0000730@163.com (H.W.); lsn_xixi@163.com (S.L.); songchunli@qqhru.edu.cn (C.S.); 2 Faculty of Health Sciences, School of Nutrition Sciences, University of Ottawa, Ottawa, ON K1H 8M5, Canada; cudenigw@uottawa.ca; 3 Zhejiang SDM Research Institute, Hangzhou 310020, China; songyuanzhang1995@163.com; 4 Department of Chemistry and Biomolecular Sciences, University of Ottawa, Ottawa, ON K1N 6N5, Canada

**Keywords:** Maillard reaction, electrostatic complex, stability, sustained release, bioaccessibility, mucus layer

## Abstract

Due to their combination of featured properties, protein and polysaccharide-based carriers show promising potential in food bioactive ingredient encapsulation, protection, and delivery. The formation of protein–polysaccharide complexes and conjugates involves non-covalent interactions and covalent interaction, respectively. The common types of protein–polysaccharide complex/conjugate-based bioactive ingredient delivery systems include emulsion (conventional emulsion, nanoemulsion, multiple emulsion, multilayered emulsion, and Pickering emulsion), microcapsule, hydrogel, and nanoparticle-based delivery systems. This review highlights the applications of protein–polysaccharide-based delivery vehicles in common bioactive ingredients including polyphenols, food proteins, bioactive peptides, carotenoids, vitamins, and minerals. The loaded food bioactive ingredients exhibited enhanced physicochemical stability, bioaccessibility, and sustained release in simulated gastrointestinal digestion. However, limited research has been conducted in determining the in vivo oral bioavailability of encapsulated bioactive compounds. An in vitro simulated gastrointestinal digestion model incorporating gut microbiota and a mucus layer is suggested for future studies.

## 1. Introduction

Food bioactive ingredients are compounds that exert health-promoting properties via modulating physiological or cellular activities, such as antioxidant, anti-inflammatory, anticancer, and immunomodulating activities [1]. Hence, development of functional foods by incorporating bioactive compounds is a promising strategy to improve human nutrition and health. However, it is challenging to simply add bioactive ingredients into food product matrices owing to the poor water solubility, low physicochemical stability, off-flavor, and limited bioaccessibility and bioavailability of many bioactive ingredients [1].

Designing suitable delivery systems for bioactive ingredients has proven to be an effective approach to overcome these shortcomings. As the two abundant food macromolecules, proteins and polysaccharides have been widely used to fabricate carriers for encapsulating bioactive ingredients [2]. Nevertheless, proteins and polysaccharides have their respective strengths and weaknesses as carrier materials. Proteins are effective at generating small emulsion droplets, but have relatively poor stability to environmental stimuli such as pH, salt, thermal, and freezing treatments. Conversely, polysaccharides possess desirable stability against environmental stresses, but have relatively poor emulsifying activity [3]. As such, the formation of protein–polysaccharide complexes would potentially combine their featured properties to overcome the shortcomings. Generally speaking, protein−polysaccharide interactions include both covalent interaction and non-covalent interactions, which can be classified into covalent protein–polysaccharide conjugates and non-covalent protein−polysaccharide complexes, respectively [1].

Covalent protein−polysaccharide conjugation can be formed using enzymatic cross-linking technique (i.e., oxidases and transglutaminase), the chemical cross-linking method (i.e., genipin, glutaraldehyde, and poly(ethylene glycol) dibutyraldehyde), and the Maillard reaction [1]. The Maillard reaction is one of the most well documented methods for preparing covalent protein–polysaccharide conjugates [4,5]. Different non-covalent interactions are responsible for the formation of protein–polysaccharide complexes depending on the distinct physicochemical properties of these biopolymers and environmental factors, such as electrostatic interactions, hydrophobic interactions, hydrogen bonding, and steric exclusion. Electrostatic interactions driven by enthalpy are a major driving force for the formation of non-covalent complexes when proteins and polysaccharides carry opposite charges [6].

In previous years, development of Maillard-type protein–polysaccharide conjugates or electrostatic complexes as delivery vehicles for food bioactive ingredients has received increasing attention [1,6]. To provide a comprehensive understanding of current research advances, this review discusses the formation and characterization of Maillard-type protein–polysaccharide conjugates and electrostatic complexes, the common types of protein–polysaccharide complex/conjugate-based delivery systems, and the applications of protein–polysaccharide complex/conjugate in encapsulation and delivery of polyphenols, food proteins, bioactive peptides, carotenoids, vitamins, and minerals.

## 2. Formation and Characterization of Maillard-Type Protein–Polysaccharide Conjugates and Electrostatic Complexes

The chemistry behind the formation of Maillard-type protein–polysaccharide conjugates and electrostatic protein–polysaccharide complexes is discussed in this section. In general, a wide range of protein sources (e.g., soy protein isolates, whey proteins, and egg white proteins) and polysaccharides (e.g., chitin, pectin, and soy hull hemicelluloses) have been applied in the production of protein–polysaccharide conjugates and complexes. The major functional properties, such as solubility, thermal stability, emulsifying and stabilizing properties, rheological and structural features, are also discussed.

### 2.1. Maillard-Type Protein–Polysaccharide Conjugates

#### 2.1.1. Formation of Covalent Protein–Polysaccharide Conjugates by Maillard Reaction

The Maillard reaction was first reported by French chemist Louis Maillard in 1912 [7]. The Maillard reaction occurs naturally under controlled pH, reaction time, temperature, and moisture conditions, and involves a series of non-enzymatic browning reactions. It starts with covalent bonding between reducing ends of carbohydrates and amino groups of proteins, especially between the ε-amino group of lysine residue and the carbonyl group of reducing carbohydrates [4,8,9]. The Maillard reaction is generally divided into early, intermediate, and final stages [4]. All three stages can occur simultaneously and are correlative [10]. Current understanding of the chemical mechanisms of formed compounds in each stage has been reviewed recently in other works [4,9] and, thus, was excluded from this review.

The most widely applied method to synthesize Maillard-type protein–polysaccharide conjugates is heat treatment, either in dry state (dry-heating method) or in aqueous solution (wet-heating method) [4]. The dry-heating method involves the heating of freeze-dried protein and polysaccharide mixture under controlled temperature (usually ranging from 40–80 °C) and relative humidity (65% or 79% most commonly used) for a duration varying from hours to weeks, which are considered as mild reaction conditions [4]. The dry-heating method has some drawbacks; it is time-consuming, costly, and with limited production scale [11]. In the wet-heating method, aqueous solution of proteins and polysaccharides is heated at a specific temperature but for a shorter time compared to the dry-heating method. Possible adverse effects during wet heating at increased temperatures are low grafting degrees with polysaccharides due to protein denaturation and polymerization [4,12]. A higher grafting degree with polysaccharides is usually positively correlated with the stronger encapsulation ability of hydrophobic bioactive compounds such as curcumin [12]. He et al. [12] developed a novel method called continuous cyclic reaction (7 cycles of low-speed agitation at 60 °C for 20 min and water-bath heating at 83 °C for 10 min) in order to increase the grafting degree of resulting SPI-dextran conjugates.

Maillard-type protein–polysaccharide conjugates exerted antimicrobial, antioxidant, and anti-antigenicity activities [13,14]. Besides these beneficial effects, advanced glycation end-products were associated with some detrimental effects including mutagenic, carcinogenic, and cytotoxic properties [4]. Optimization of reaction parameters (i.e., time, pH, water activity, and temperature) may prevent the generation of the antinutritional and toxic compounds by controlling the reaction progress [15]. For example, egg white-galactomannan conjugates were generated in a controlled dry state (79% relative humidity) at 60 °C for two weeks, and their safety was confirmed by mammalian cell proliferation assay [16]. By and large, glycated proteins fabricated by the Maillard reaction are safer than chemically modified proteins [15]. Therefore, Maillard-type protein–polysaccharide conjugates have the potential to be safely utilized in food products.

#### 2.1.2. Characterization of Maillard-Type Protein–Polysaccharide Conjugates

Various techniques have been used to assess the formation and physicochemical properties of protein–polysaccharide conjugates, such as molecular weight profile, particle size distribution, browning index, free amino groups, and structural characteristics [4]. Specifically, sodium dodecyl sulfate polyacrylamide gel electrophoresis (SDS-PAGE) is commonly used to detect molecular weight changes after conjugate formation. For instance, the occurrence of a protein band with a high molecular weight (on the top of separating gel) indicated the formation of β-lactoglobulin–gum Acacia Seyal conjugates [17]. Mass spectrometry has also been applied to confirm conjugate formation by analyzing the increment in molecular mass [4]. Desorption ionization-time of flight mass spectrometry (MALDI-TOF MS) analysis was used to demonstrate that bovine serum albumin (BSA) was glycated with chitin oligosaccharides at 43% relative humidity and 60 °C after 6 and 12 h [18]. In addition, the particle size distribution of protein–polysaccharide conjugates is often determined by dynamic light scattering (DLS). DLS measurements showed that the average particle size of lysozyme–pullulan conjugates was 3.5-fold higher and the range of particle size distribution was broader compared to that of untreated lysozyme [19].

Since browning always occurs during the Maillard reaction, a browning index can be used to determine the extent of the reaction between proteins and polysaccharides by measuring absorbance of the conjugates at 420 nm [17,20,21]. A free amino group of protein–polysaccharide conjugates is used as an indicator of degree of substitution during the Maillard reaction [22], and is usually determined by o-phthaldialdehyde (OPA) assay or 2,4,6-trinitrobenzenesulfonic acid (TNBS) method. When β-lactoglobulin and gum Acacia seyal were reacted at 60 °C and 79% relative humidity, ~30% decrease of free amino groups was observed after 12 h due to conjugation with the polysaccharide [17].

The secondary structures of conjugates are commonly analyzed by circular dichroism (CD) and Fourier-transform infrared spectroscopy (FTIR). CD spectroscopy revealed that the secondary structures of lysozyme changed after conjugation to pullulan. Compared with native lysozyme, the conjugates had reduced α-helical structure (from 33% to 24%) and increased β-turn (from 2% to 9%) and random coil (from 29% to 33%) [19]. Similarly, substantial changes were reported in the CD spectrum of WPI-polysaccharide conjugates compared to native WPI [22]. FTIR is another relevant technique for investigating the structure and interaction of protein–polysaccharide conjugates based on alterations in the spectra, such as the appearance of new peaks and changes in the peak location and intensity [23]. FTIR analysis suggested that conjugation of soybean protein isolate with glucose or chitosan oligosaccharide decreased the contents of α-helix and β-sheet with a concomitant increase in β-turn and random coil [24]. Lastly, changes in protein conformations due to conjugation with polysaccharides could be monitored by measuring the intrinsic fluorescence of Trp [19]. The occurrence of a red shift phenomenon may result from a more hydrophilic microenvironment surrounding protein molecules after glycosylation, which leads to alterations in protein conformations [25]. Reduction of fluorescence intensity after conjugation was observed in several studies, which has been attributed to protein conformational changes and the strong steric-hindrance effect of the polysaccharide chain that shields the fluorescence signal of Trp residues [19,23,26].

#### 2.1.3. Functional Properties of Maillard-Type Protein–Polysaccharide Conjugates

##### Solubility and Thermal Stability

Solubility is one of the most important factors that determine the other functional properties of biopolymers, such as thermal stability and emulsifying properties [25]. Generally, the water solubility of Maillard-type protein–polysaccharide conjugates could be significantly enhanced compared to untreated proteins [4]. Ma et al. [25]. reported that the solubility of soy protein isolate (SPI)-pectin conjugates was significantly improved compared to native SPI. Conjugates of whey proteins (β-lactoglobulin, α-lactalbumin, and BSA) and dextran (molecular weight of 10 and 20 kDa) exhibited increased solubility at the isoelectric point of the crude proteins [27]. The increased solubility was mainly attributed to the grafted hydrophilic polysaccharide moieties, as well as the reduced intermolecular aggregation of protein molecules owing to the steric-repulsion effect induced by the polysaccharide [8,25]. However, contradictory results have been reported. Reduction in the solubility of egg white protein–pectin conjugates was observed with increased Maillard reaction time [28]. The biochemical complexity of proteins, different natures of polysaccharides, and formation of intermolecular disulfide bonds during conjugation may result in this discrepancy [4].

Improved thermal stability of glycosylated proteins produced by the Maillard reaction has been widely reported [4,29]. For instance, soy hull hemicelluloses-SPI conjugates exhibited a higher thermal stability compared to the individual biopolymer which was determined by the thermal gravimetric analysis. Specifically, the conjugates containing the SPI contents from 20% to 60% showed higher thermal stability. The authors suggested that the increased protein content in the conjugates was positively associated with the enhanced thermal stability [29].

##### Emulsifying and Stabilizing Properties

Among their functional properties, the emulsifying properties of protein–polysaccharide conjugates are the most extensively investigated [4]. It is generally reported that protein–polysaccharide conjugates possess better emulsifying properties than crude proteins at both low and neutral pHs, particularly at a pH close to the isoelectric point [8]. Critical parameters, such as the molecular weight and structure of the polysaccharide, reaction time, and ratio of protein and polysaccharide, play important roles in the emulsifying and stabilizing properties of the conjugates [4,8]. For example, with a reaction time of 12 h, milk protein isolate/κ-carrageenan conjugates at a ratio of 1:1 at 65 °C were utilized to effectively produce a stable oil-in-water emulsion during storage at 40 °C for 2 d [30]. Moreover, the balanced amphiphilic nature of protein–polysaccharide conjugates is indispensable for enhancing their emulsifying properties [4].

Ma et al. [25]. recently demonstrated the emulsifying activity index of the SPI-pectin conjugates had a 3-fold increase compared with the native SPI [25]. The improved emulsifying activity was attributed to inhibition of protein–protein interactions resulting from glycosylation [31]. Ultrasound treatment contributes to further enhancement in the emulsifying activity of conjugates [31,32]. The increased degree of graft, surface hydrophobicity, and extended spatial conformations of proteins induced by ultrasound were responsible for the improved emulsifying properties [32]. In contrast, emulsifying properties were shown to decrease in BSA-glucose and BSA-mannose conjugates compared to BSA, partly due to the decreased surface hydrophobicity and alterations in conformational flexibility [33].

Likewise, the emulsifying stability of protein–polysaccharide conjugates is higher than that of native proteins [25,29,34]. Compared to native SPI, SPI-citrus pectin conjugates prepared by dry-heating conditions showed a 2-fold increase in the emulsifying stability index [25]. This possibly resulted from the strong steric-hindrance effect from polysaccharides, which effectively prevented the oil droplets from re-coalescence [31,34]. Moreover, ultrasound-assisted reaction increased the emulsifying stability index 2-fold compared to SPI-citrus pectin conjugates formed by the traditional wet-heating method [32]. This was mainly owing to the fact that ultrasound treatment changes the surface hydrophobicity and secondary structures of protein molecules [31]. On the other hand, mild ultrasound treatment (100 W) favored the control of the Maillard reaction to produce myofibrillar protein–dextran conjugates with enhanced emulsifying ability and stability when compared to conjugates generated with high-intensity ultrasound (300 W) [35].

### 2.2. Electrostatic Protein–Polysaccharide Complexes

#### 2.2.1. Formation of Electrostatic Protein–Polysaccharide Complexes

**Figure 1:**
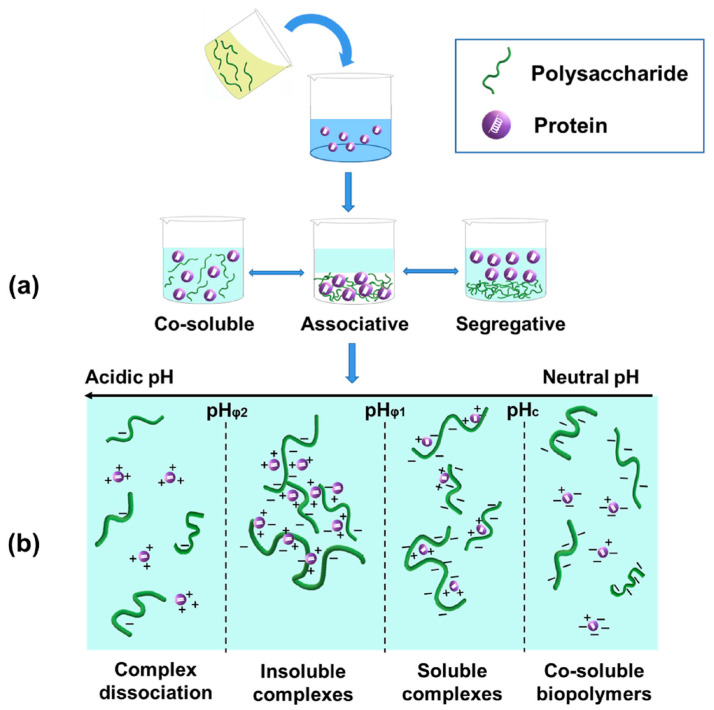
(**a**) Formation of different phase systems between proteins and polysaccharides, including co-soluble biopolymers, associative phase separation (complex coacervation), and segregative phase separation (thermodynamic incompatibility). (**b**) A schematic diagram of the transitions of protein–polysaccharide complexes induced by pH changes.

As shown in Figure 1a, a biopolymer mixture of protein and polysaccharide may result in different phase systems, including co-soluble polymers, associative phase separation (complex coacervation) and segregative phase separation (thermodynamic incompatibility), which highly depends on factors such as pH, ionic strength, biopolymer concentration, and protein/polysaccharide ratio [36]. Biopolymers are co-soluble and remain stable in dilute solutions due to the dominating effects of mixing entropy [36,37]. However, the mixture has a tendency to be unstable with an increase in biopolymer concentration. This may lead to two phase behaviors that largely depend upon the electrostatic interaction between biopolymers [36]. When proteins and polysaccharides carry a similar net charge, segregative phase behavior (separation into protein-rich phase and polysaccharide-rich phase) may occur due to electrostatic repulsion [36]. On the other hand, electrostatic attraction results in the occurrence of associative phase behavior (complex coacervation) when two biopolymers exhibit the opposite net charge [37]. Complex coacervation is a liquid–liquid phase separation phenomenon where one phase is rich in biopolymers and the other phase is rich in solvent. Coacervation contributes to the formation of electrostatic complexes between oppositely charged proteins and polysaccharides [38]. Overall, the main driving force for the complexation is electrostatic interaction when proteins and polysaccharides carry opposite charges.

The formed complexes can be either soluble or insoluble, depending on various parameters, especially pH (depicted in Figure 1b) [38,39]. According to the distinct structure-forming characteristics of complexes, four critical pH values have been defined [40]. The first critical pH value (pH_c_) has been regarded as the onset of the formation of soluble complexes, which is the first detectable interaction. The pH_c_ of electrostatic protein–polysaccharide complexes is generally near or below the isoelectric point (pI) of the protein since all naturally occurring polysaccharides are neutral or acidic except chitosan [40,41]. When pH is higher than pH_c_, proteins and polysaccharides are co-soluble molecules in solution [40]. With a decrease in pH from pH_c_, solubility of the complexes decreases and they start to aggregate into insoluble forms due to charge neutralization at the second critical pH (pH_φ1_), which results in a sharp increase in turbidity. The maximum turbidity value is reached at pH_opt_, which is the electric neutral points of proteins and polysaccharides. As the pH reduces further and reaches the pH_φ2_, the complexes disassociate into individual biopolymers because reactive sites on the polysaccharide chains are more protonated [40,42,43,44]. Taking the electrostatic complexes between BSA and sodium alginate as an example, the critical pH values (pH_c_, pH_φ1_, pH_opt_, and pH_φ2_) were 4.8, 4.2, 2.8 and 1.8, respectively [45].

Besides pH, other parameters that influence the formation of electrostatic protein–polysaccharide complexes include ionic strength, charge density and distribution, polysaccharide type, biopolymer mixing ratio, and total concentration, temperature, and shearing rate. These factors have been extensively discussed in a recent review article [36] and, thus, further details will not be provided in this section. Moreover, protein–polysaccharide complexation is able to modify functional attributes compared to the individual component such as enhanced protein water solubility, emulsifying and stabilizing properties, as well as foaming ability and stability, which have been reviewed recently [36]. Thus, this review article mainly focused on their rheological and structural properties.

#### 2.2.2. Rheological and Structural Characteristics of Electrostatic Protein–Polysaccharide Complexes

Rheological properties of protein–polysaccharide complexes have gained increasing attention as they play important roles in determining the application of biopolymer complexes in food products [46]. To comprehensively understand the rheological properties of biopolymer complex coacervates, storage modulus (G′), loss modulus (G″), loss tangent (tan δ), critical value of stress (τc) at the linear viscoelastic (LVE) region, fracture stress and strain, and crossover point need to be determined [47]. For example, a recent study used small amplitude oscillatory shear (SAOS) to extensively investigate the rheological properties of coacervates of rice bran protein–flaxseed gum [47]. Among these rheological parameters, G′ and G″ are commonly measured in strain sweep or frequency sweep tests [44,48].

Rheological characteristics of biopolymer complex coacervates are significantly influenced by environmental factors, such as pH, protein/polysaccharide ratio, and ionic strength [44,47,48]. Hasanvanda and Rafeb [47] explored the influence of different pH values (3.3, 4.0, and 5.3) and protein/polysaccharide ratios (3:1, 6:1, and 9:1) on the rheological properties of rice bran protein–flaxseed gum coacervates. At pH 4.0 and biopolymer ratio 9:1, the coacervates showed significantly higher structural strength (G′LVE 10,200 Pa) and loss modulus (G″LVE 2130 Pa) as determined by amplitude sweep at 25 °C [47]. In general, low salt concentration could cause salt-enhanced effects whereas the salt-reduced effect may occur with further increase in salt concentration [44]. Specifically, where the frequency ranged from 0.1 to 100 rad/s, G′ values of β-lactoglobulin–pectin coacervates increased from ~103 Pa to 105 Pa as the ionic strength increased from 0.01 to 0.21 M, while a further increase in ionic strength to 0.41 M decreased the G′ values below 103 Pa, because high salt concentration weakened the binding between β-lactoglobulin and pectin [44].

Furthermore, rheology data are often used to indicate the structures of protein–polysaccharides coacervates. Specifically, a higher G′ value than G″ of coacervates, such as rice bran protein–flaxseed gum coacervates and β-lactoglobulin–pectin coacervates, indicates the formation of highly interconnected gel-like structures [44,47]. Also, other analytical techniques are broadly used to investigate the microstructure of complex coacervates. Cryo-scanning electron microscopy (Cryo-SEM) study suggested that whey protein isolate (WPI)–*Tremella fuciformis* polysaccharide (TP) complexes exhibited more ordered structures than each of the two biopolymers [49]. CD spectroscopy indicated the α-helix contents of WPI–TP complexes increased when compared to free WPI [49]. Likewise, Fourier transform infrared (FTIR) spectroscopy revealed that complexation with polysaccharides, including carrageenan, chitosan and sodium alginate, resulted in changes of the secondary structures of gelatin, i.e., the content of collagen-like triple helices in an α-chain increased [50]. In 2018, Xu et al. [51] utilized small angle X-ray scattering (SAXS) and small angle neutron scattering (SANS) to understand the effects of polysaccharide charge pattern on the microstructures of β-lactoglobulin–pectin complex coacervates [51]. SAXS and SANS data indicated that compact primary particles are the major building blocks of complex coacervates, which are formed by overlapping β-lactoglobulin-binding pectin chains and bridged by protein-rich clusters. It was revealed that the size and distribution of the protein-rich clusters were influenced by the charge densities of pectin. More importantly, changes in the spatial arrangements of the primary particles could result in the complex coacervates transforming into precipitates. This study proposed the possibility of modifying the microstructure of protein–polysaccharide complex coacervates by utilization of polysaccharides with distinct charge patterns [51]. On the other hand, protein types could also influence the structures of complex coacervates. Compared to gelatin–gum arabic complex coacervates, soy protein–gum arabic complex coacervates were less tight and structured with a characteristic length scale of 40 nm according to the Doi-Onuki model [46]. Jin et al. [52] recently reported that pulsed electric field (PEF) treatment changed the structures of α-amylase-pectin electrostatic complexes to branched, ring, or circles-like shapes. To achieve these effects, it is possible the PEF technique modified the charge distribution of proteins and polysaccharides and subsequently influenced their electrostatic interactions and complex coacervation.

The chemistry behind the formation, commonly investigated structural characteristics and functional properties of Maillard-type protein–polysaccharide conjugates and electrostatic complexes discussed in this section were summarized in Table 1.

## 3. Different Types of Protein–Polysaccharide Complex- or Conjugate-Based Delivery Systems

Protein–polysaccharide complexes or conjugates can be utilized as building blocks to fabricate delivery systems with more complex structures, such as emulsion, microcapsule, hydrogel, and nanoparticle-based delivery systems, which were discussed in this review [1]. Their common preparation methods, features and nature of the commonly encapsulated compounds are summarized in Table 2.

### 3.1. Emulsion-Based Delivery Systems

Protein–polysaccharide complexes or conjugates are widely used to fabricate emulsion-based delivery systems due to their enhanced emulsifying stability and better protection for the encapsulated compounds compared to individual proteins or polysaccharides [1,53]. Protein–polysaccharide complexes or conjugates are commonly used as emulsifiers to generate different types of emulsions including conventional emulsions, nanoemulsions, multiple emulsions, multilayered emulsions and Pickering emulsions [1].

#### 3.1.1. Conventional O/W Emulsions

**Figure 2:**
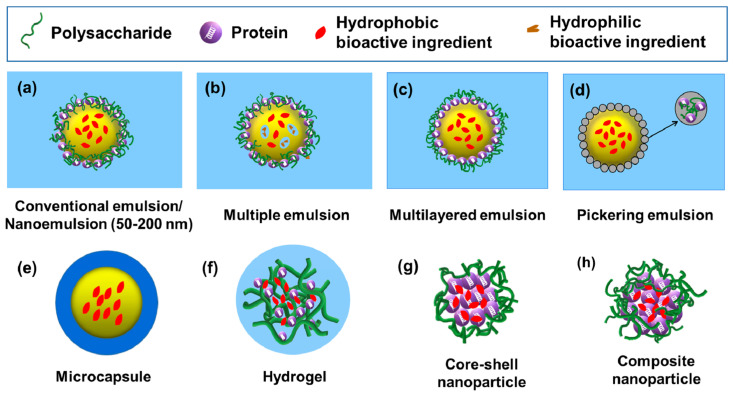
Different types of protein–polysaccharide complex/conjugate-based delivery systems for bioactive ingredients. (**a**) Conventional emulsion/Nanoemulsion (50–200 nm); (**b**) Multiple emulsion; (**c**) Multilayered emulsion; (**d**) Pickering emulsion; (**e**) Microcapsule; (**f**) Hydrogel; (**g**) Core-shell nanoparticle; (**h**) Composite nanoparticle.

Conventional emulsions have the mean droplet radii in the range of 0.2–100 µm (Figure 2a), which are thermodynamically unstable systems and prone to be optically turbid or opaque [54]. They can be formed using a high-shear mixer or a high-pressure homogenizer [54,55]. Oil-in-water (O/W) emulsions are commonly prepared for encapsulation of lipophilic nutraceutical compounds. Both protein–polysaccharide complexes and conjugates showed increased ability to stabilized the emulsion droplets against unfavorable environmental conditions [55,56]. For example, the O/W emulsion prepared by casein–chitosan complexes possessed good stability in a broad pH range from 3.5 to 6.5 [55]. Additionally, the good stability of corn oil-in-water emulsions formulated with pea protein isolate–gum arabic conjugates was due to their small particle size, high surface charge and strong steric hindrance [56].

#### 3.1.2. Nanoemulsions (O/W)

Nanoemulsions typically have mean droplet radii that range from 50–200 nm (Figure 2a), and are thermodynamically stable isotropic systems that tend to be transparent or slightly opaque [54]. Nanoemulsions are developed for improving the delivery of bioactive compounds mainly due to their small droplet sizes and particle shapes dispersed in the continuous phase [57]. Nanoemulsions are fabricated using high-energy or low-energy methods. The high-energy method involves mechanical devices, such as high-speed blenders, high-pressure homogenizers, microfluidizers and ultrasonic probes. The low-energy methods include phase inversion and solvent mixing approaches [54]. Nanoemulsions produced from soy protein–soy polysaccharide complexes exhibited long-term stability at pH values of 2–8 and 0.2 M NaCl [58] or after heat treatment (80 °C for 60 min) [59], indicating strong potential of the complexes to function as nanoscale carriers for delivering lipophilic bioactive ingredients.

#### 3.1.3. Multiple Emulsions

Multiple emulsions are complex poly-dispersed systems that simultaneously contain both oil-in-water and water-in-oil emulsions [60]. Water-in-oil-in water (W_1_/O/W_2_) emulsion is the most common type of multiple emulsions, which comprises of small water droplets within larger oil droplets that are dispersed in an aqueous continuous phase (Figure 2b) [1,61]. Due to the presence of both water and oil compartments, multiple emulsions can simultaneously encapsulate and deliver hydrophilic and lipophilic bioactive compounds [1,62]. Both protein–polysaccharide complexes and Maillard-type conjugates have been applied to stabilize multiple emulsions, which have strong potential to function as bioactive ingredient delivery systems owing to their enhanced encapsulation efficiency, physical stability, protection and controlled release properties of loaded compounds [62,63,64]. Moreover, it was reported that pectin-whey protein complexes can be utilized as a desirable emulsifier with comparable properties as small molecule surfactants (Tween 80) for stabilizing W_1_/O/W_2_ emulsions [65].

#### 3.1.4. Multilayered Emulsions

As shown in Figure 2c, multilayered emulsions are characterized as oil droplets electrostatically stabilized by a multilayered interfacial membrane. Generally, the interfacial membrane is composed of an emulsifier (e.g., proteins) and a charged biopolymer (e.g., polysaccharides) [66]. The multilayered emulsion structures are generated using the layer-by-layer (LbL) electrostatic deposition technique [54]. For example, the multilayered O/W emulsions stabilized by protein–polysaccharide complexes can be fabricated by direct adsorption of oppositely charged polysaccharides on a primary layer of proteins surrounding the oil droplet surface [66]. Multilayered emulsions have exhibited good physical stability to environmental stresses (e.g., ionic strength, pH and temperatures) and provided a promising delivery system for food bioactives [66,67]. For instance, multilayered emulsions formulated with β-lactoglobulin–pectin complex have been used for delivery purposes [67].

#### 3.1.5. Pickering Emulsions

Pickering emulsions are stabilized by solid particles that are irreversibly adsorbed to the oil–water interface, as illustrated in Figure 2d [54]. Rotor-stator homogenization, high-pressure homogenization and sonication are the most commonly used techniques for preparing Pickering emulsions [68]. The solid particles function as a mechanical (steric) barrier that provide long-term physical stability of Pickering emulsions against coalescence and Ostwald ripening. In order to effectively stabilize the Pickering emulsions, the average size of particles at the interface needs to be at least 10–100 times smaller than that of emulsion droplets [54]. Protein–polysaccharide particles, such as xanthan gum–zein complex and ovotransferrin–gum arabic complex particles, have been recently applied as emulsifiers to fabricate food-grade Pickering emulsions with promising features as carriers for the protection and delivery of bioactive compounds [69,70].

### 3.2. Microcapsule-Based Delivery Systems

As depicted in Figure 2e, microcapsules consist of a membrane shell that creates a reservoir to encapsulate the core material [71]. Microcapsules are often designed for the protection and delivery of hydrophobic bioactive ingredients, such as oils and fat-soluble vitamins [1,72,73,74]. Moreover, microcapsules generated using double emulsion prior to the complex coacervation method have been used successfully to encapsulate hydrophilic compounds, such as anthocyanins, and improve their stability under harsh processing and storage conditions [75]. The shell materials of microcapsules have been devised using both Maillard-type protein–polysaccharide conjugates and electrostatic protein–polysaccharide complexes [72,73]. For example, lycopene-loaded microcapsules have been constructed using Maillard-type whey protein isolate–xylo-oligosaccharide conjugates as the shell material [69]. In contrast, chia seed oil-loaded microcapsules were prepared by using chia seed protein–gum complexes as shell materials, leading to better release and digestive properties of the encapsulated oils compared to microcapsules derived from using individual protein or polysaccharide as shell materials [72].

### 3.3. Hydrogel-Based Delivery Systems

Hydrogels are three-dimensional networks that are formed by polymer cross-linking through physical, ionic, or covalent interactions, which can entrap large amounts of water (Figure 2f) [76,77]. The common method to prepare protein–polysaccharide hydrogels is complex coacervation and the subsequent thermal treatment to induce gelation [76]. The thermal process was reported to enhance the stability of hydrogels under different environmental stimuli and achieve sustained release of loaded food bioactives [78]. On the other hand, hydrogels can be produced by the assembly of Maillard-type protein–polysaccharide conjugates, which also possess good stability and dispersibility [9].

Based on particle size, hydrogels can be divided into microgels (d. µm 1–350) and nanogels (d. nm 20–250) [79]. Protein–polysaccharide-based nanogels are promising delivery vehicles for bioactive ingredients owing to their high loading capacity, controlled release property, improved bioaccessibility, good chemical stability, and smart responses to environmental stimuli [1]. Hydrogels are devised to mainly encapsulate hydrophilic compounds (e.g., folic acid and riboflavin) but they can also deliver hydrophobic bioactives (e.g., curcumin) [80,81,82].

### 3.4. Nanoparticle-Based Delivery Systems

Core-shell structure is one of the most common morphologies of protein–polysaccharide hybrid nanoparticles, as shown in Figure 2g. To produce core-shell nanoparticles, particles are first formed by creating protein nanoparticles as the inner core, followed by coating the protein core with hydrophilic polysaccharide as the shell structure [1]. Core-shell nanoparticles are often manufactured to encapsulate and deliver hydrophobic bioactive compounds (e.g., curcumin) due to the mostly hydrophobic interactions occurring with hydrophobic proteins such as zein [1,83]. Overall, the protein inner core provides good protection for the encapsulated compounds whereas the polysaccharide shell layer prevents particle aggregation and enhances stability by generating strong steric and electrostatic repulsions [1].

Composite nanoparticles are generated by formation of the protein–polysaccharide complexes prior to loading of bioactive compounds, as illustrated in Figure 2h [1,84]. It was demonstrated that hydrophobic interaction, electrostatic interaction, and hydrogen bonding played vital roles in the formation of zein–propylene glycol alginate composite nanoparticles. These composite nanoparticles functioned as a promising β-carotene delivery system by improving the physicochemical stability and controlled release of the hydrophobic compound [84]. Recently, Chen et al. reported that modification of zein–chitosan composite nanoparticles by atmospheric cold plasma treatment increased the encapsulation efficiency and dispersion stability of loaded resveratrol compared to untreated nanoparticles. The increased encapsulation efficiently was ascribed to the enhanced interaction between zein and chitosan after atmospheric cold plasma treatment [85].

## 4. Applications of Protein–Polysaccharide Complexes/Conjugates as Delivery Systems for Food Bioactive Ingredients

**Figure 3:**
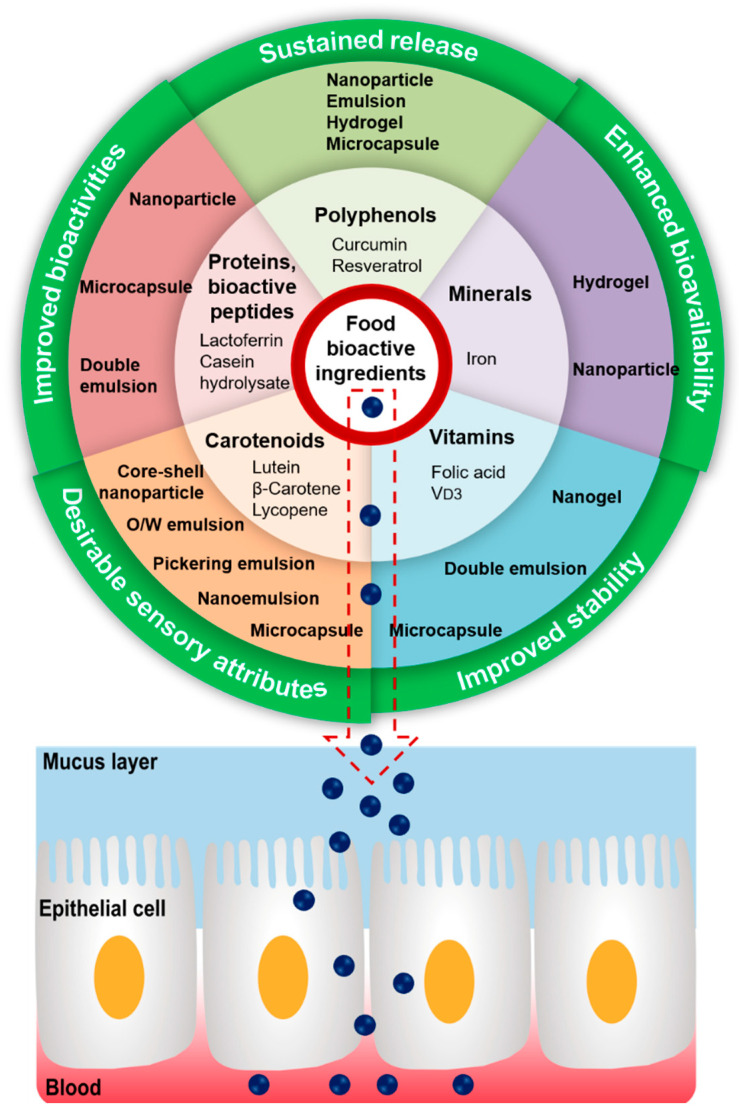
Summary of key points of discussion in Section 4. Considering the improved properties of loaded food bioactive ingredients, protein–polysaccharide-based delivery vehicles are promising approaches for enhancing cellular uptake and achieving systematic circulation.

Food bioactive ingredients commonly loaded in the protein–polysaccharide-based delivery vehicles include polyphenols, proteins, bioactive peptides, carotenoids, vitamins, minerals, and essential oils [1]. The most widely investigated compounds were selected as representative bioactive ingredients, and major research findings on these compounds are summarized in Table 3 and illustrated in Figure 3. A recent review provided a detailed discussion on the microencapsulation of essential oils by the complex coacervation method using protein and polysaccharide [86]; thus, this topic is not reiterated in this section.

### 4.1. Polyphenols

Polyphenols have been simply classified into flavonoids and non-flavonoids [129]. A wide range of flavonoid-type polyphenolic compounds, such as anthocyanin [75,130], quercetin [131,132], isoquercetin [133], quercetagetin [134], epigallocatechin gallate [135], and curcumin [136], have been successfully encapsulated into different protein–polysaccharide-based carriers for protection, sustained release, and delivery.

Curcumin is often used as the model of hydrophobic bioactive compounds when designing and fabricating novel delivery systems. Hence, this review focused on only recent research progress on the protein–polysaccharide-based delivery systems for curcumin. Likewise, resveratrol was selected for discussion as the representative non-flavonoid polyphenol.

#### 4.1.1. Curcumin

Curcumin, also called diferuloylmethane, is a natural polyphenolic compound present in the rhizome of *Curcuma longa* (turmeric) and in other *Curcuma* spp. [137]. Due to its wide range of health-promoting activities, such as antimutagenic, antimicrobial, anti-inflammatory, and antioxidant activities, curcumin has strong potential to be applied as a functional food ingredient and nutraceutical [54]. However, curcumin has poor water solubility, low stability, and limited bioavailability, which restrict its application in the food industry. Substantial research efforts have been made to develop food-grade curcumin delivery vehicles in order to overcome the challenges and effectively deliver curcumin in targeted physiological sites [54]. Different types of curcumin delivery systems have been fabricated using protein–polysaccharide conjugates or complexes as building blocks, including core-shell nanoparticle, composite nanoparticle, microcapsule, emulsion, and hydrogel-based delivery systems.

In the past decade, a wide range of protein–polysaccharide complexes have been designed to fabricate core-shell nanoparticles for curcumin delivery, such as casein–soy polysaccharide [87], pea protein–carboxymethylated corn fiber gum [89], cationized gelatin and sodium alginate [92], insect protein–chitosan [91], native and succinylated pea protein–chitosan [138], whey protein–gum arabic [93], and soybean protein isolate–fucoidan complexes [136]. Encapsulation efficiencies of curcumin in these developed core-shell nanoparticles ranged from 30–99% [89,91,93]. The curcumin-loaded casein–soy polysaccharide nanoparticles showed long-term dispersion stability after 30 days of storage at 25 °C [87]. Likewise, the chemical, thermal, and photo stabilities of encapsulated curcumin have been significantly improved. Specifically, lysozyme–*A. Sphaerocephala Krasch* polysaccharide complex nanoparticles increased curcumin stability at physiological pH in aqueous buffer [88]. Approximate 75% of free curcumin degraded in phosphate buffer within 6 min, while 59% and 46% of encapsulated curcumin remained stable after 24 h and 48 h incubation, respectively [88]. Compared to free curcumin (15%), curcumin-loaded pea protein–carboxymethylated corn fiber gum nanoparticles showed a significantly higher thermal stability (95%) after heat treatment (80 °C, 30 min, pH 3.5) [89]. Regarding photo stability, it was reported that after 90 min of UV radiation, the residual levels of curcumin in the free and nanoencapsulated forms (pea protein isolate–high methoxyl pectin complexes) were 4% and 34%, respectively [90]. In addition, the release profile and oral bioavailability of encapsulated curcumin are of great significance for achieving its health-promoting activities. The release kinetics of curcumin from insect protein–chitosan nanoparticles were determined under the simulated oral, gastric, and intestinal conditions [91]. More than 90% of encapsulated curcumin was released after the simulated digestion process, including 6.3% in oral phase, 8.2% in gastric phase, and 78.1% in intestinal phase. A recent study demonstrated that the oral bioavailability of curcumin loaded in casein–soy polysaccharide complexes increased 3.4-fold in blood of mice compared to the curcumin/Tween 20 treatment [87]. Furthermore, encapsulated curcumin in core-shell nanoparticles showed better antioxidant and anticancer activities in vitro compared to free curcumin [88,89,92,93].

Likewise, encapsulation of curcumin in protein–polysaccharide composite nanoparticles has gained significant research attention. The possible encapsulation mechanism of these nanoparticles is that the formation of protein–polysaccharide complexes results in protein unfolding and exposure of the hydrophobic pockets, which facilitate curcumin binding to the protein moiety of complexes via hydrophobic interactions [94]. Encapsulation efficiencies of curcumin in composite nanoparticles are usually higher than 80% [97,139]. Moreover, curcumin-encapsulated composite nanoparticles have shown great potential in food applications owing to their high dispersion stability and color stability [140]. For example, curcumin-loaded composite nanoparticles (whey protein isolate–sodium alginate nanocomplex) possessed acceptable dispersion stability (no obvious precipitates) in model food processing and storage conditions, such as high concentrations of sucrose and NaCl, and heat treatment at 90 °C for 2 h [95]. Composite nanoparticles effectively provided curcumin protection against light and different pH [94,95,96]. A sustained release of curcumin from composite nanoparticles has been observed in simulated gastric and intestinal fluids, which led to enhanced bioaccessibility of curcumin [96]. Taking the curcumin-loaded zein–fucoidan nanoparticle as an example, the cumulative release rates of curcumin were 10% and 62% in simulated gastric fluid (90 min) and simulated intestinal fluid (240 min), respectively [97]. Many studies have demonstrated that the in vitro antioxidant activities of curcumin in composite nanoparticles were remarkably improved [95,98].

Besides nanoparticle-based delivery systems, curcumin has been successfully loaded in other types of protein–polysaccharide delivery vehicles, such as oil-in-water emulsions [99,100], microcapsules [101], and hydrogels [102]. Specifically, the curcumin loading efficiency of nanoemulsion stabilized by casein–soy soluble polysaccharide complexes was as high as 99.9% and only 3% of the loaded curcumin degraded during storage at 4 °C for 40 days [99]. A controlled release of curcumin from the nanoemulsion was achieved during simulated gastrointestinal digestion and an 11-fold increase in curcumin oral bioavailability in mice was observed [99]. Likewise, nanoemulsion with Maillard-type bovine serum albumin–dextran conjugates was fabricated for protection and oral delivery of curcumin [100]. When curcumin was encapsulated in spray-dried microcapsules fabricated with whey protein–maltodextrin and gum arabic, it became resistant to in vitro gastric digestion but was released in simulated intestinal fluids [101]. Recently, Su et al [102]. developed a β-lactoglobulin–propylene glycol alginate-based hydrogel for co-delivery of curcumin and probiotics. Besides protection of probiotics, the encapsulated curcumin had a sustained release in simulated gastrointestinal tract conditions and exhibited good stability when exposed to light and during long-term storage [102].

#### 4.1.2. Resveratrol

Resveratrol is a non-flavonoid polyphenol with numerous health promoting properties, such as antioxidant, anti-inflammatory, anti-proliferative, anticancer, and anti-aging activities [106]. Nonetheless, utilization of resveratrol as a nutraceutical or functional food ingredient is challenged by its poor water solubility, chemical instability, and low bioavailability [106]. To address these issues, distinct types of protein–polysaccharide-based delivery systems, such as core-shell nanoparticles, oil-in-water emulsions, and multilayered emulsions, have been developed [104,108,141].

When resveratrol was loaded into core-shell nanoparticles, the encapsulation efficiencies often ranged from 50% to 90% [105,142]. It was reported that 28/40 dual-frequency ultrasound effectively increased the encapsulation efficiency of resveratrol in zein–chitosan complex nanoparticles from 51% to 65% [142]. After encapsulation, resveratrol lost its crystalline structure and changed to the amorphous form in alginate/chitosan–zein nanoparticles and α-lactalbumin–chitosan nanoparticles [103,104]. The major driving forces between resveratrol and α-lactalbumin–chitosan nanoparticles include hydrophobic interaction and hydrogen bonding [103]. Light, heat, and storage stabilities of encapsulated resveratrol in core-shell nanoparticles were remarkably increased compared to those of free resveratrol. For example, after exposure to UV light for 200 min and heat treatment at 85 °C for 300 min, the retention rates of free and encapsulated resveratrol in α-lactalbumin–chitosan nanoparticles were 44% and 47%, and 85% and 86%, respectively [103]. Moreover, sustained in vitro release of resveratrol from nanoparticles in simulated gastrointestinal digestion could be enhanced. For instance, in simulated gastric phase, 77% of free resveratrol was released compared to 52% released from resveratrol encapsulated in zein nanoparticles [104]. A recent study evidently demonstrated that compared to free resveratrol, the in vitro bioaccessibility of encapsulated resveratrol in hollow zein–chitosan nanoparticles increased 2-fold from 44% to 90% [105]. Consequently, in vitro antioxidant and anticancer activities of the encapsulated resveratrol were improved as well [103,106]. However, there is a dearth of information on the oral bioavailability and in vivo bioactivities of encapsulated resveratrol.

It has been reported that when loading a low amount of resveratrol (0.02 g/100 g) into the oil-in-water emulsion stabilized by Maillard-type sodium caseinate–corn starch hydrolysate conjugates, the in vitro antioxidant activity significantly increased [107]. Food-grade protein–polysaccharide multilayered emulsions have also been designed to encapsulate and protect resveratrol and to increase its antioxidant activity [108]. Lactoferrin–alginate multilayered emulsions were reported to be stable only at a high concentration of alginate (>0.18% *w*/*w*) owing to the bridging flocculation effect at low alginate concentrations [108]. The antioxidant activity of this resveratrol-loaded multilayered emulsions was maintained during storage for 4 weeks whereas decreased antioxidant activity of free resveratrol was observed in the third week [108].

### 4.2. Proteins and Bioactive Peptides

Beyond their nutritional properties, several food proteins and peptides have demonstrated numerous health-promoting properties, such as antihypertensive, antimicrobial, cholesterol-lowering, antithrombotic, anticancer, immunomodulatory, mineral binding, opioid-like, and antioxidant activities [143]. However, the in vitro biological activities of proteins and bioactive peptides do not generally translate into in vivo pharmacological functions in animal studies and human clinical trials [2]. One of the major reasons for this discrepancy is the low biostability or bioaccessibility of proteins and peptides during gastrointestinal digestion, which further results in low bioavailability [144,145]. In addition, bioactive peptides often have a bitter taste and hygroscopicity due to the exposure of hydrophobic and hygroscopic amino acid residues resulting from hydrolysis, which limit their applications in food product development [2]. Protein–polysaccharide-based delivery systems have been developed for protection and controlled release of proteins and bioactive peptides in order to enhance their in vivo bioactivities, and sensory and physicochemical properties. For example, lactoferrin has been trapped in nanocarriers for broadening its applications in food and pharmaceutical industries [109]. The highest encapsulation efficiency of lactoferrin in whey protein isolate–high methoxyl pectin nanoparticles was reported at the optimum condition of 2:1 protein–pectin ratio (*w*/*w*) and pre-acidification at pH 3.5. However, encapsulation efficiency was only 25% at the optimized conditions [109]. In addition to enhancing the encapsulation efficiency, the release profile, stability, and biological activities of encapsulated lactoferrin need to be explored in future studies.

Furthermore, a soybean protein isolate–pectin microcapsule has been designed to encapsulate casein hydrolysates for attenuating the bitter taste and hygroscopicity [110]. The encapsulation efficiency decreased from 92% to 79% when the loading amount of casein hydrolysate increased from 50% to 150% (*w*/*w*). The results showed that encapsulated hydrolysates had lower hygroscopicity and less bitter taste compared to free hydrolysate [110]. Jo and Schaaf [111] recently fabricated food-grade double emulsions (W_1_/O/W_2_) to improve the controlled release of bioactive peptides at different temperatures. The bioactive peptide–polysaccharide complex-loaded double emulsions had encapsulation efficiency of >90% and possessed a higher heat stability. Controlled release of encapsulated bioactive peptide from the double emulsions was observed at 45 °C (<1%) and 65 °C (<30%) during storage for 4 h. Oil types played notable roles in the peptide release from the double emulsions. More rapid release of the peptide was observed for double emulsions containing oil with medium chain triglycerides, e.g., coconut oil, compared to oil with long chain triglycerides, e.g., canola oil [111].

### 4.3. Carotenoids

Carotenoids are natural pigments in various fruits and vegetables, which have many human health benefits, such as antioxidant, intercellular communication, and immune system activities. Carotenoids can be classified into two groups on the basis of their chemical structures, including xanthophylls (e.g., lutein) and carotenes (e.g., β-carotene and lycopene) [146]. It is challenging to utilize carotenoids as natural colorants in food products due to their low water solubility and chemical instability. Encapsulation in protein–polysaccharide systems is a suitable approach to overcome this barrier.

#### 4.3.1. Lutein

Core-shell nanoparticle-based carriers are widely investigated for encapsulation and oral delivery of lutein [112]. For example, compared to lutein-loaded protein nanoparticles, modified rice protein–carboxymethylcellulose nanoparticles efficiently controlled the release of lutein during gastrointestinal digestion, effectively inhibited the proliferation of breast cancer cells, and increased the lutein uptake rate and absorption [112]. Nonetheless, proteins also play essential roles in the formation of core-shell nanoparticles for lutein delivery. It was suggested that a high mass ratio of protein–lutein increased encapsulation efficiency. The encapsulation efficiency of lutein in zein–soluble soybean polysaccharide nanoparticles was higher than 80% when the mass ratio of zein–lutein was 25:1. However, encapsulation efficiency was only 35% at the mass ratio of 10:1 [113]. Bioaccessibility of the encapsulated lutein was two times higher than that of free lutein [113]. To increase the stability of lutein carriers, the formation parameters of whey protein isolate–pectin nanoparticles (protein–polysaccharide ratio, pH, and type of pectin) have been optimized. The most stable system was established with low methoxyl pectin at a protein–polysaccharide ratio of 4:1 and pH 5.0; the carrier remained stable after storage for 30 days [114].

Oil-in-water emulsions are another common type of lutein delivery system with good stability, which can be emulsified by both Maillard-type protein–polysaccharide conjugates and electrostatic complexes [115,116]. Specifically, lutein-loaded emulsions stabilized by casein–dextrin conjugates were reported to be stable at a wide range of pH values (from 3 to 7) and not aggregate during simulated gastric digestion; this was attributed to the steric repulsion resulting from the dextran [115]. Moreover, lutein-enriched emulsions stabilized by egg yolk-modified starch complexes, especially egg yolk–hydroxypropyl distarch phosphate complexes, showed good physical stability, low lipid oxidation, and high lutein retention during storage at 37 °C [116].

Lutein has also been encapsulated in Pickering emulsions stabilized by β-lactoglobulin–gum arabic-based nanoparticles. The nanoparticles exhibited a core-shell structure and significantly contributed to the stability of the Pickering emulsions. The formed emulsions showed a high resistance against flocculation and coalescence and favorable storage stability. After 12 weeks of storage, more than 90% of encapsulated lutein was retained in the Pickering emulsions [117].

#### 4.3.2. β-Carotene

Due to the antioxidant and pro-vitamin A nature of β-carotene, many attempts have been made to develop delivery systems to enhance its dispersant state, chemical stability, bioavailability, and functionalities. By and large, O/W emulsions are effective for the protection and delivery of β-carotene [118,147]. O/W emulsion-based β-carotene delivery systems are commonly stabilized by Maillard-type protein–polysaccharide conjugates [118,119,120,147]. The increased emulsifying activity of protein–polysaccharide (e.g., soy protein isolate–*Pleurotus eryngii* polysaccharide) conjugates was attributed to their decreased surface hydrophobicity and flat surface morphology [119]. A recent study demonstrated that ovalbumin–dextran conjugates possessed good emulsifying stability in different environmental conditions, including pH (3.0–10.0), high ionic strength (150 mM NaCl), and thermal treatment (90 °C for 30 min) [147]. Bioaccessibility of encapsulated β-carotene in O/W emulsions stabilized by deamidated wheat gluten–maltodextrin conjugates was close to 60% [118]. The enhanced bioaccessibility favorably contributed to the increased antioxidant activity of β-carotene in Caco-2 intestinal cells [119,120].

β-carotene has been successfully entrapped in O/W nanoemulsions stabilized by whey protein hydrolysate–pectin soluble complexes and the concentration of β-carotene was considered as a critical parameter [121]. Average droplet size of the nanoemulsion was ~95 nm, and encapsulation efficiency was as high as 92% when the concentration of β-carotene was 25 mg/100 g emulsion. However, when the concentration of β-carotene increased to 75 mg/100 g emulsion, the nanoemulsion displayed a larger droplet size (127 nm) and a significantly lower encapsulation efficiency (27%). Additionally, lower concentration of loaded β-carotene increased nanoemulsion stability against droplet coalescence and retarded the loss of antioxidant activity of β-carotene during storage [121]. Moreover, Yi et al. [122] designed high-internal phase Pickering emulsions stabilized by pea protein–high methoxyl pectin colloidal particles as novel β-carotene delivery systems. The spherical protein–polysaccharide colloidal particles were formed spontaneously by electrostatic interaction. The fabricated β-carotene-loaded Pickering emulsions displayed high stability against pH variation. However, bioaccessibility of the encapsulated β-carotene in Pickering emulsions was only 26% [122], which needs to be improved if intended to be used in practical food applications.

#### 4.3.3. Lycopene

Due to its highly unsaturated structure, lycopene is sensitive to heat and light, which may result in oxidation and isomerization [123]. Protein–polysaccharide-based microcapsules have been fabricated to encapsulate lycopene [123]. When gelatin–pectin complexes were utilized as the wall materials, encapsulation efficiency of lycopene was higher than 90%. However, this microcapsule-based delivery system did not provide effective protection for lycopene during storage, with degradation rate of 14% per week [123]. When Maillard-type whey protein isolate–xylo-oilgosaccharide conjugates were applied as wall materials, storage stability of the encapsulated lycopene was improved. The degradation rates of lycopene after storage for 36 days at 4, 25, and 40 °C were 12%, 54%, and 60%, respectively. Meanwhile, the microcapsules based on protein–polysaccharide conjugates resulted in high encapsulation efficiency (94%) and lycopene solubility (92 g/L). Compared to free lycopene, bioaccessibility of the encapsulated lycopene significantly increased from 16% to 60%. Hence, whey protein isolate–xylo-oilgosaccharide conjugate-based microcapsules are considered as promising lycopene delivery systems [73].

### 4.4. Vitamins

Vitamins are defined as a group of essential micronutrients that cannot be synthesized by the human body; they are classified into fat-soluble (A, D, E, and K) and water-soluble vitamins (e.g., folic acid) [148]. Deficiency of vitamins can result in severe diseases, such as scurvy and night blindness [148]. Vitamins can easily be degraded during food processing and storage since they are chemically reactive and sensitive to environmental factors such as light, pH, temperature, and oxygen [148]. It is well established that microencapsulation and nanoencapsulation prevent vitamin loss during food processing and storage, and help to achieve targeted delivery and sustained release [148,149]. However, limited research has been conducted to date on development of protein–polysaccharide-based vitamin delivery systems. Most existing research has particularly focused on folic acid and vitamin D_3_ delivery.

To improve the stability and controlled delivery of folic acid, soy protein–soy polysaccharide complex nanogels were developed [81]. The folic acid-loaded nanogels possessed good water dispersibility in acidic conditions due to the presence of a polysaccharide surface. More importantly, the nanogels provided strong protection of folic acid from heat, oxygen, and light in acidic conditions, whereas the encapsulated folic acid showed a rapid release at neutral pH value [81]. Another study prepared and optimized stable W_1_/O/W_2_ whey protein–maltodextrin double emulsions for folic acid encapsulation by the low-energy emulsification technique [124]. The folic acid-encapsulated nanoemulsions showed potential for utilization in fortification of liquid foods but limited applications in solid foods [124]. To address this drawback, a spray drying technique was used to prepare folic acid-incorporated whey protein–pectin nanoparticles, which led to the lowest release rate of folic acid at pH 4 and highest release at pH 11 [125].

Vitamin D_3_ is a lipid-soluble compound that easily degrades under acidic conditions. Ovalbumin–pectin nanocomplexes were developed as effective carriers for vitamin D_3_ with encapsulation efficiency of 96%. Encapsulation of vitamin D_3_ in the nanocomplexes was driven by electrostatic interactions, hydrogen bonding, and hydrophobic interactions. In vitro release study indicated that only 11% of loaded vitamin D_3_ was released from the nanocomplexes in simulated gastric fluid within 60 min, whereas in simulated intestinal fluid, the cumulative release rate within 120 min reached 98% [126]. Furthermore, it was demonstrated that the addition of sodium alginate significantly enhanced the stability of vitamin D_3_-incorporated ovalbumin–pectin nanocomplexes due to the strong negative charge of sodium alginate [150].

### 4.5. Mineral (Iron)

Some minerals, such as iron, calcium, and zinc, play important biological roles and are essential micronutrients for maintaining human health. Hence, food fortification with minerals has been considered as one of the most effective strategies for combating micronutrient malnutrition globally. However, mineral fortification can adversely influence the physical and sensory properties of foods, and the absorption and bioavailability of fortified minerals could be impeded by other food components such as phytates [151]. To overcome these challenges, research efforts have led to the development of effective protein–polysaccharide-based carriers for protection and delivery of minerals, especially iron [127,128]. Kazemi-Taskooh and Varidi [127] designed a composite cold-set hydrogel formulated with whey protein isolate and gellan gum as an iron delivery system. The encapsulation efficiency of iron in hydrogel reached 94%, and was affected by total biopolymer concentration, protein/polysaccharide ratio, and iron concentration. However, a majority of the encapsulated iron (up to 89%) was released from the hydrogel in simulated gastric digestion rather than simulated intestinal digestion. This could be because of the cationic net charge of proteins at low acidic pH, resulting in electrostatic repulsion and dissociation of bound iron from the complex. Increase gastric stability and sustained release of iron in the intestinal phase need to be enhanced by modification of the hydrogel structures. On the other hand, nanoparticle-based iron delivery systems, which were fabricated with whey protein isolate and gum arabic, dramatically slowed the release of entrapped iron (only 20% released) in the simulated gastric phase [128].

## 5. Conclusions and Future Perspectives

Protein–polysaccharide complexes and conjugate-based carriers have shown tremendous promise for encapsulation, protection, and delivery of food bioactive ingredients. The loaded food bioactive ingredients exhibited enhanced physicochemical stability, bioaccessibility, and sustained release in simulated gastrointestinal conditions. However, it is still challenging to achieve the optimum delivery for specific bioactive ingredients, for example, simultaneously optimizing all parameters such as physicochemical stability, loading amount, controlled release, good protection, bioaccessibility, and bioavailability. Considering the different characteristics of specific bioactive ingredients, the overall performance of the delivery systems could be improved by selection of suitable proteins and polysaccharides, and control of operation parameters of formation of protein–polysaccharide conjugates and complexes.

Currently, most studies have applied an in vitro simulated gastrointestinal digestion model that consists of digestive juices and enzymes to evaluate the release profile and bioaccessibility of encapsulated bioactive ingredients, without taking into account the role of gut microbiota. An in vitro simulator of the human intestinal microbial ecosystem (SHIME) is a potential model to address this challenge. More importantly, mucus can be incorporated into the SHIME model, which can be utilized to investigate the impact of the mucus layer on cellular uptake and transport mechanisms of bioactive-loaded delivery systems. Besides in vitro studies, more research needs to be conducted in understanding the effect of protein–polysaccharide-based delivery systems on in vivo oral bioavailability of encapsulated bioactive compounds. Moreover, current research has mainly focused on the application of the protein–polysaccharide-based delivery systems to polyphenols, particularly curcumin. Future research needs to focus on the design and fabrication of efficient protein–polysaccharide-based delivery vehicles for vitamins and minerals.

## Figures and Tables

**Table 1:** Comparison between Maillard-type protein–polysaccharide conjugates and electrostatic complexes.

	Maillard-Type Protein–Polysaccharide Conjugates	Electrostatic Protein–Polysaccharide Complexes
Formation	Covalent bonding between reducing ends of carbohydrates and amino groups of proteins [4,8,9,10]	Different phase systems between proteins and polysaccharides, including co-soluble biopolymers, complex coacervation, and thermodynamic incompatibility [36]
Structural characteristics	Secondary structures analyzed by CD [19,22] and FTIR [23,24]	Secondary structures analyzed by CD [49] and FTIR [50]; Microstructures analyzed by Cryo-SEM [49], SAXS and SANS [51]
Functional properties	Enhanced functional properties compared to native proteins: water solubility [25,27]; thermal stability [29]; emulsifying property [25,31,32]; emulsifying stability [25,29,34]	Rheological properties [46,47,48]

**Table 2:** Overview of protein–polysaccharide complex/conjugate-based delivery systems for bioactive ingredients.

Types	Common Preparation Methods	Features	Nature of Commonly Encapsulated Compounds	References
Conventional O/W emulsions	High-energy methods (high-shear mixers or high-pressure homogenizers)	Mean droplet radii (0.2–100 μm); thermodynamically unstable systems	Lipophilic	[53,54,55,56]
Nanoemulsions (O/W)	High-energy methods (high-speed blenders, high-pressure homogenizers, microfluidizers or ultrasonic probes); Low-energy methods (phase inversion and solvent mixing approaches)	Mean droplet radii (50–200 nm); thermodynamically stable isotropic systems	Lipophilic	[54,57,58,59]
Multiple emulsions (W_1_/O/W_2_)	Producing primary W/O emulsions before generating W_1_/O/W_2_ emulsions	Presence of both water and oil compartments	Hydrophilic and lipophilic	[60,61,62,63,64,65]
Multilayered emulsions (O/W)	Layer-by-layer (LbL) electrostatic deposition technique	Stabilized by a multilayered interfacial membrane; good physical stability to environmental stresses	Lipophilic	[54,66,67]
Pickering emulsions (O/W)	High-energy methods (Rotor-stator homogenization, high-pressure homogenization, sonication)	Stabilized by solid particles; long-term physical stability	Lipophilic	[54,68,69,70]
Microcapsules	Emulsion-spray drying; double emulsion–complex coacervation method	Containing a membrane shell	Lipophilic	[71,72,73,74,75]
Hydrogels	Complex coacervation and thermal treatment to induce gelation	Three-dimensional networks; polymer crosslinking through physical, ionic or covalent interactions; including microgels (d. μm 1–350) and nanogels (d. nm 20–250)	Hydrophilic	[76,77,78,79,80,81,82]
Core-shell nanoparticles	Coating protein nanoparticles with polysaccharides	Including protein inner core and polysaccharide shell layer	Lipophilic	[1,83]
Composite nanoparticles	Anti-solvent precipitation; emulsification–evaporation method	Formation of the protein–polysaccharide complexes prior to loading of bioactive compounds	Lipophilic	[1,84,85]

**Table 3:** Applications of protein–polysaccharide complexes/conjugates as delivery systems for representative food bioactive ingredients.

Bioactive Ingredient	Composition of Delivery System ^1^	Type of Delivery System	Improved Properties of Encapsulated Bioactive Ingredient	References
Polyphenols				
Curcumin	Casein-soy polysaccharide	Core-shell nanoparticle	Long-term dispersion stability; oral bioavailability	[87]
Curcumin	Lysozyme-*A. Sphaerocephala Krasch* polysaccharide; pea protein–carboxymethylated corn fiber gum; pea protein isolate–high methoxyl pectin	Core-shell nanoparticle	Chemical, thermal, and photo stabilities	[88,89,90]
Curcumin	Insect protein–chitosan	Core-shell nanoparticle	Release profile	[91]
Curcumin	Cationised gelatin–sodium alginate; whey protein nanofibril–gum arabic	Core-shell nanoparticle	In vitro antioxidant and anticancer activities	[92,93]
Curcumin	Whey protein isolate–sodium alginate; ovalbumin–κ-carrageenan	Composite nanoparticle	Dispersion, light and chemical stabilities	[94,95,96]
Curcumin	Zein–fucoidan	Composite nanoparticle	Sustained release	[97]
Curcumin	Lactoferrin–pectin	Composite nanoparticle	In vitro antioxidant activities	[98]
Curcumin	Casein–soy soluble polysaccharide	Nanoemulsion	Storage stability; controlled release; oral bioavailability	[99]
Curcumin	Bovine serum albumin–dextran conjugate	Nanoemulsion	Chemical stability; oral bioavailability	[100]
Curcumin	Whey protein–maltodextrin and gum arabic	Microcapsule	Sustained release	[101]
Curcumin	β-lactoglobulin–propylene glycol alginate	Hydrogel	Sustained release; light and storage stabilities	[102]
Resveratrol	α-lactalbumin–chitosan;	Core-shell nanoparticle	Light, heat and storage stabilities	[103]
Resveratrol	Zein–alginate/chitosan; zein–chitosan	Core-shell nanoparticle	Sustained release; bioaccessibility; storage stability	[104,105]
Resveratrol	Zein–pectin; α-lactalbumin–chitosan	Core-shell nanoparticle	In vitro antioxidant and anticancer activities	[103,106]
Resveratrol	Sodium caseinate–corn starch hydrolysate conjugate	O/W emulsion	In vitro antioxidant activities	[107]
Resveratrol	Lactoferrin–alginate	Multilayered emulsion	In vitro antioxidant activity	[108]
Proteins and bioactive peptides				
Lactoferrin	Whey protein isolate–high methoxyl pectin	Nanoparticle	Not determined	[109]
Casein hydrolysate	Soybean protein isolate–pectin	Microcapsule	Attenuated bitter taste; decreased hygroscopicity	[110]
Bioactive peptide	Bioactive peptide–pectin/chitosan	Double emulsion	Controlled release	[111]
Carotenoids				
Lutein	Modified rice protein–carboxymethylcellulose	Core-shell nanoparticle	Controlled release; inhibited the proliferation of breast cancer cells; increased the lutein uptake rate and absorption	[112]
Lutein	Zein–soluble soybean polysaccharide	Core-shell nanoparticle	Bioaccessibility	[113]
Lutein	Whey protein isolate–pectin	Core-shell nanoparticle	Storage stability	[114]
Lutein	Casein–dextrin conjugate	O/W emulsion	Dispersion stability	[115]
Lutein	Egg yolk–modified starch	O/W emulsion	Physical and storage stabilities; low lipid oxidation	[116]
Lutein	β-lactoglobulin-gum arabic	Pickering emulsion	Storage stability	[117]
β-Carotene	Soy protein isolate–*Pleurotus eryngii* polysaccharide conjugate; wheat gluten–maltodextrin/citrus pectin conjugate; oat protein isolate–*Pleurotus ostreatus* β-glucan conjugate	O/W emulsion	Bioaccessibiliy; in vitro antioxidant activity	[118,119,120]
β-Carotene	Whey protein hydrolysate–pectin	Nanoemulsion	Storage stability; in vitro antioxidant activity	[121]
β-Carotene	Pea protein–high methoxyl pectin	Pickering emulsion	pH stability	[122]
Lycopene	Gelatin–pectin	Microcapsule	No desirable storage stability	[123]
Lycopene	Whey protein isolate–xylo-oilgosaccharide conjugate	Microcapsule	Storage stability; bioaccessibility	[73]
Vitamins				
Folic acid	Soy protein–soy polysaccharide	Nanogel	Water dispersibility at acidic conditions; chemical, light and heat stabilities	[81]
Folic acid	Whey protein–maltodextrin	Double emulsion	Not determined	[124]
Folic acid	Whey protein–pectin	Double emulsion	Sustained release	[125]
Vitamin D_3_	Ovalbumin–pectin	Microcapsule	Sustained release	[126]
Mineral				
Iron	Whey protein isolate–gellan gum	Hydrogel	Burst release in simulated gastric digestion	[127]
Iron	Whey protein isolate–gum arabic	Nanoparticle	Sustained release	[128]

^1^ Only protein–polysaccharide conjugates were identified, otherwise they were protein–polysaccharide complexes.
